# Severe immunosuppression and not a cytokine storm characterizes COVID-19 infections

**DOI:** 10.1172/jci.insight.140329

**Published:** 2020-09-03

**Authors:** Kenneth E. Remy, Monty Mazer, David A. Striker, Ali H. Ellebedy, Andrew H. Walton, Jacqueline Unsinger, Teresa M. Blood, Philip A. Mudd, Daehan J. Yi, Daniel A. Mannion, Dale F. Osborne, R. Scott Martin, Nitin J. Anand, James P. Bosanquet, Jane Blood, Anne M. Drewry, Charles C. Caldwell, Isaiah R. Turnbull, Scott C. Brakenridge, Lyle L. Moldwawer, Richard S. Hotchkiss

**Affiliations:** 1 Department of Pediatrics,; 2 Department of Internal Medicine, and; 3 Department of Anesthesiology, Washington University School of Medicine in St. Louis, St. Louis, Missouri, USA.; 4 Department of Critical Care, Missouri Baptist Medical Center, St. Louis, USA.; 5 Department of Pathology and Immunology, and; 6 Department of Emergency Medicine, Washington University School of Medicine in St. Louis, St. Louis, Missouri, USA.; 7 Saint Louis University School of Medicine, St. Louis, Missouri, USA.; 8 Department of Surgery, University of Cincinnati College of Medicine, Cincinnati, Ohio, USA.; 9 Department of Surgery, Washington University School of Medicine in St. Louis, St. Louis, Missouri, USA.; 10 Department of Surgery, Sepsis and Critical Illness Research Center, University of Florida College of Medicine, Gainesville, Florida, USA.

**Keywords:** COVID-19, Adaptive immunity

## Abstract

COVID-19–associated morbidity and mortality have been attributed to a pathologic host response. Two divergent hypotheses have been proposed: hyperinflammatory cytokine storm; and failure of host protective immunity that results in unrestrained viral dissemination and organ injury. A key explanation for the inability to address this controversy has been the lack of diagnostic tools to evaluate immune function in COVID-19 infections. ELISpot, a highly sensitive, functional immunoassay, was employed in 27 patients with COVID-19, 51 patients with sepsis, 18 critically ill nonseptic (CINS) patients, and 27 healthy control volunteers to evaluate adaptive and innate immune status by quantitating T cell IFN-ɣ and monocyte TFN-α production. Circulating T cell subsets were profoundly reduced in COVID-19 patients. Additionally, stimulated blood mononuclear cells produced less than 40%–50% of the IFN-ɣ and TNF-α observed in septic and CINS patients, consistent with markedly impaired immune effector cell function. Approximately 25% of COVID-19 patients had increased IL-6 levels that were not associated with elevations in other canonical proinflammatory cytokines. Collectively, these findings support the hypothesis that COVID-19 suppresses host functional adaptive and innate immunity. Importantly, IL-7 administered ex vivo restored T cell IFN-ɣ production in COVID-19 patients. Thus, ELISpot may functionally characterize host immunity in COVID-19 and inform prospective therapies.

## Abstract 2

ELISpot, a highly sensitive, functional immunoassay, suggests that COVID-19 is immunosuppressive and lacks substantial cytokine storm.

## Graphical abstract

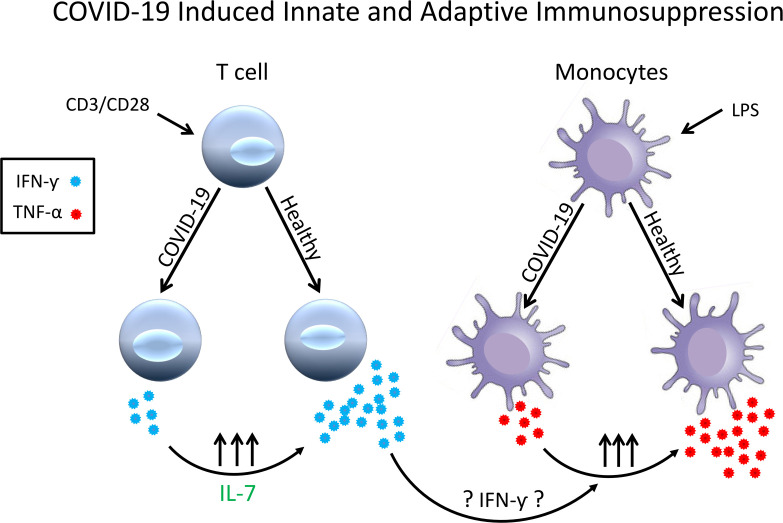

## Introduction

One of the most remarkable realities about the current SARS–CoV-2 infection outbreak (COVID-19) is that despite intense worldwide investigations, the decisive pathophysiologic processes that are responsible for patient morbidity and mortality remain unknown. Currently, the predominant paradigm is that an overexuberant immune response mediated by excessive proinflammatory cytokines drives excessive lung injury and a procoagulant state (1–7). Accordingly, death is assumed to be primarily due to inflammatory lung injury, disturbances in micro- and macrocirculation, and resultant respiratory failure or vascular coagulopathy (8–14). This concept of a cytokine storm–mediated death in COVID-19 patients has been popularized in both the lay press and many leading scientific publications (6, 15). Based on this theory, a number of anti-cytokine and antiinflammatory therapies are being tested in COVID-19, including anti–IL-6(R) antibodies, IL-1 receptor antagonists, and JAK/STAT inhibitors, with early trial results failing to demonstrate significant efficacy (2, 3, 9, 15–18).

Paradoxically, a second and diametrically opposed theory for COVID-19–induced morbidity and mortality is an *“*immunologic collapse” of the host’s protective system (15, 19–21). This collapse of host protective immunity manifests itself as a failure to control unrestrained viral replication and dissemination with direct host cytotoxicity. Support for this contrasting theory is based on the observed progressive and profound lymphopenia, often to levels seen in patients with AIDS (22). Multiple recent studies show that unlike the cytokine storm, which is often considered episodic, lymphopenia is incessant in critically ill COVID-19 patients with and correlates with increased secondary infections and death (11, 13). Postmortem studies of patients dying of COVID-19 have also described a devastating loss of immune cells in spleen and secondary lymphoid organs (23). Multiple lymphocyte subsets are lost, including CD4^+^ T, CD8^+^ T, and NK cells, which play vital antiviral roles, and in B cells, which are essential for making antibodies that neutralize the virus (4, 21, 24–26).

Personalized medicine approaches require a better understanding of which of these immune endotypes predominate, because the appropriate intervention is diametrically different depending upon whether the patient is experiencing hyperinflammation or profound immunosuppression. For example, anti–IL-6(R) antibodies, IL-1 receptor antagonists, and JAK/STAT inhibitors are currently undergoing clinical testing in patients with COVID-19 (27–32) and carry the potential to further compromise the patient’s ability to eradicate the virus. Conversely, treatment with immune stimulants such as checkpoint inhibitors, IL-7, IFN-γ, and GM-CSF, currently either proposed or in active clinical trials in COVID-19 (15, 33), could exacerbate a dysfunctional and robust inflammatory response and worsen organ injury.

Two distinct and key questions must be addressed in critically ill COVID-19 patients: (i) what is their primary immune endotype, i.e., hyperinflammatory versus immunosuppressive? and (ii) how does each evolve over time with regard to disease progression or resolution. A better understanding of the COVID-19 patient’s immune status would be instrumental in guiding proper immunotherapy.

There have been many efforts to determine patient immune endotype using genomic or proteomic biomarkers of immunity (34, 35). While these methods have been helpful in predicting outcomes in sepsis and other disorders (36, 37), in general they have either not been able to provide an accurate assessment of the functional state of host immunity, as it varies over time, or have been used to determine response to therapy. Enzyme-linked immunosorbent spot (ELISpot) is a highly sensitive, functional immunoassay that measures the number of cytokine-secreting cells at the single-cell level in response to ex vivo stimulation (38, 39). A key advantage of ELISpot is that the assay has excellent dynamic range. ELISpot can detect as few as 1 in 100,000 cytokine-secreting cells. Furthermore, ELISpot can test simultaneously the integrity and robustness of the 2 disparate arms of immunity, i.e., innate (blood monocytes and low-density granulocytes) and adaptive cellular immunity (blood lymphocytes) by focusing on the responses of individual cell populations to cell-specific agonists.

The purpose of this study was to determine whether critically ill COVID-19 patients have an exaggerated proinflammatory cytokine storm versus an immunosuppressive immunological endotype, and determine whether there are changes in immune function during disease progression. To provide a comprehensive evaluation, we used conventional flow cytometry to quantitate the effect of COVID-19–mediated depletion of immune effector cells. In addition to quantitating circulating pro- and antiinflammatory cytokines, we evaluated adaptive and innate immune systems via serial ELISpot assays of T cell IFN-ɣ and monocyte TNF-α production, respectively.

## Results

### Demographic and clinical characteristics.

We enrolled 27 patients with COVID-19, 51 patients with sepsis, 18 critically ill nonseptic (CINS) patients in a prospective observational cohort study (Table 1) evaluating innate and adaptive immune function in SARS–CoV-2 infection more than 2 weeks after intensive care unit (ICU) admission. Primary diagnosis for COVID-19, sepsis, and CINS are included in Supplemental Tables 1–4; supplemental material available online with this article; https://doi.org/10.1172/jci.insight.140329DS1 Twenty-seven healthy participants served as controls.

**Figure 1:**
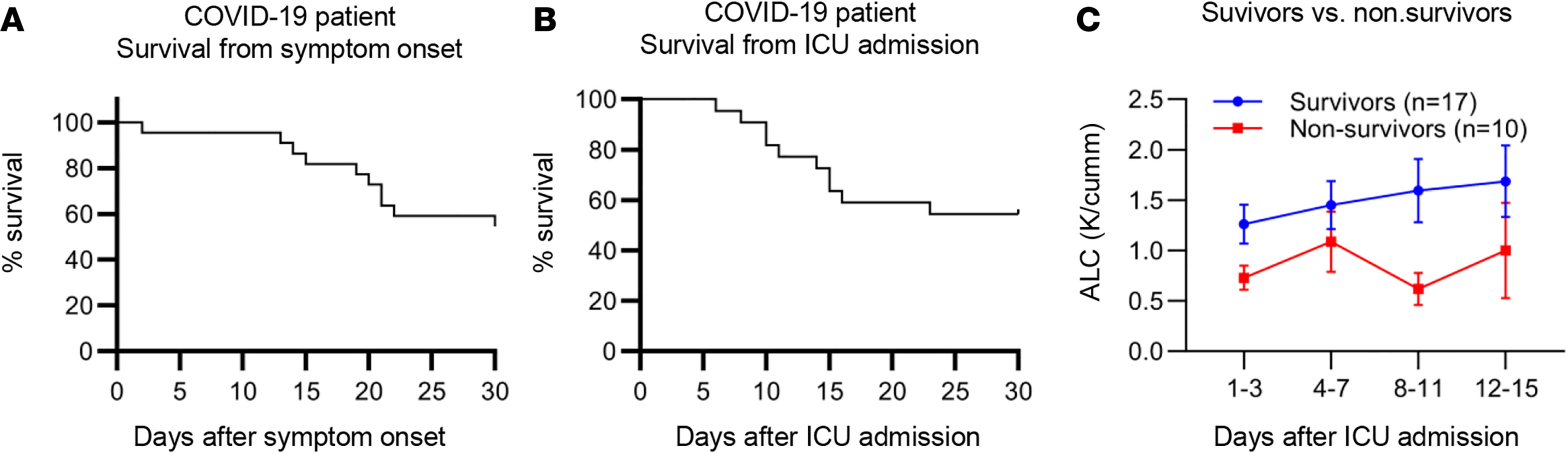
COVID-19 patient survival. Survival is plotted as a function from symptom onset (**A**) and ICU admission (**B**). Difference in ALC over time between survivors and nonsurvivors (**C**). Total patients *n* = 27; survivors *n* = 17, nonsurvivors *n* = 10.

COVID-19 patients were hospitalized in the ICU with a mean of 6 (range 1–14) days after onset of symptoms. Twenty-three of 27 COVID-19 patients were intubated and received invasive mechanical ventilation on average 1 (range 0–5) day from ICU admission. The mean sequential organ failure assessment (SOFA) and APACHE II scores were the equivalent in the COVID-19 and sepsis cohorts (7 and 18, respectively). The 30 day mortality was greater in the COVID-19 group than in patients with sepsis (37% vs. 22%; *P* = 0.14), but the difference did not reach statistical significance. All nonsurviving COVID-19 patients died more than 2 weeks after onset of symptoms and at least 6 days following admission to the ICU (Figure 1, A and B).

The absolute lymphocyte counts (ALC) for COVID-19 patients was 900 cells/mm^3^, and nonsurvivors had persistent lymphopenia throughout the course of illness compared with COVID-19 survivors (Figure 1C and Table 1). Ten of the 27 COVID-19 patients (37%) had evidence of secondary infections during the first 30 days after enrollment. Thirty percent of patients with secondary infection were nonsurvivors, and 1 patient had coinfection with coronavirus 229E at admission.

### Plasma cytokines.

**Figure 2:**
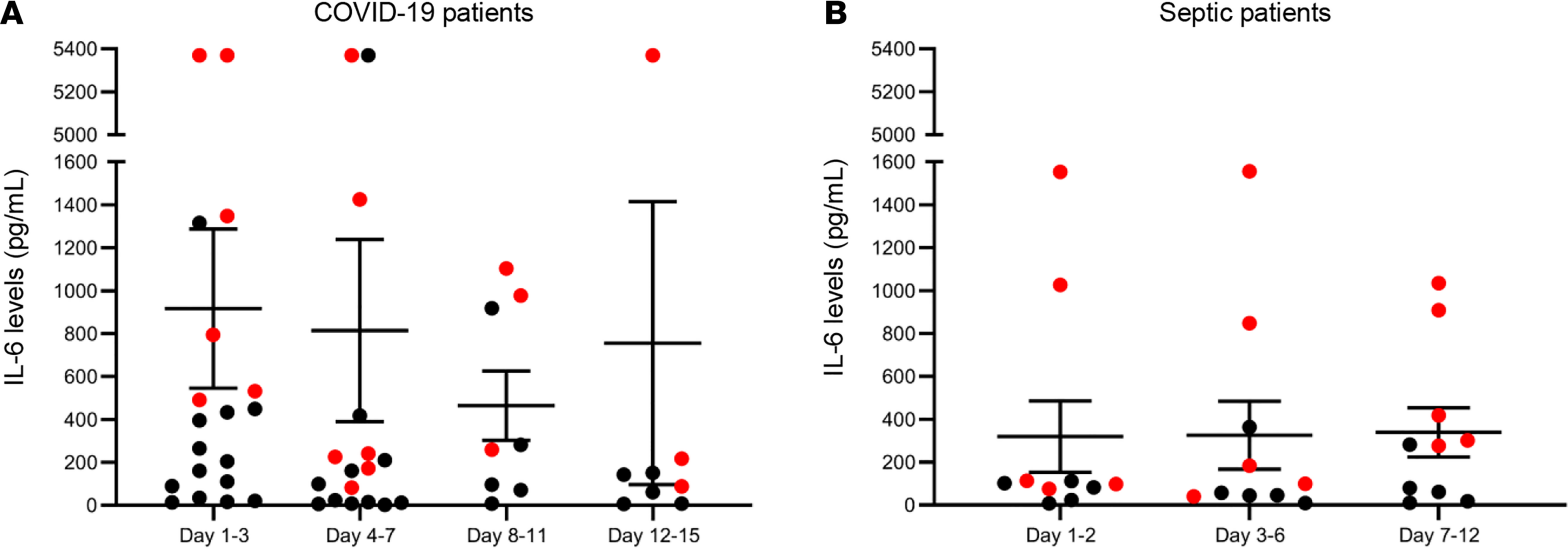
Plasma IL-6 levels in patients with COVID-19 and sepsis. Dot plot representing plasma IL-6 levels for COVID-19 patients (**A**) and patients with sepsis (**B**) at various time points after ICU admission. Data bars represent mean ± SEM. Red dots represent nonsurvivors. Septic patients *n* = 10; COVID-19 days 1–3 *n* = 19, days 4–7 17, days 8–11 *n* = 8, days 12–15 *n* = 8.

To evaluate the inflammatory response over time, we measured plasma cytokines in COVID-19, septic, and CINS patients and healthy control participants (Table 2). Patients with COVID-19 and sepsis patients were followed for up to 4 serial time points after ICU admission. The mean number of sample time points was 2.2 for the COVID-19 patients and 3 for septic patients. A single time point was used for healthy controls and CINS patients. Of note, for COVID-19 patients, the blood sample for cytokine analysis was obtained within the first 24 hours from clinical deterioration (endotracheal intubation) after admission to the ICU in order to try to capture the early hyperinflammatory phase of infection. Although several key proinflammatory cytokines, including IL-1β, IFN-ɣ, and TNF-α, were modestly increased in COVID-19 patients compared with healthy control participants, the increases were near the lower limit of detection of the assay (Table 2). There was considerable variation in plasma IL-6 levels in COVID-19 patients, with a range from 6 to more than 5000 pg/μL (Figure 2). IL-6 concentrations were elevated compared with those in healthy controls.

### COVID-19 induces profound suppression of T cell IFN-ɣ production.

**Figure 3:**
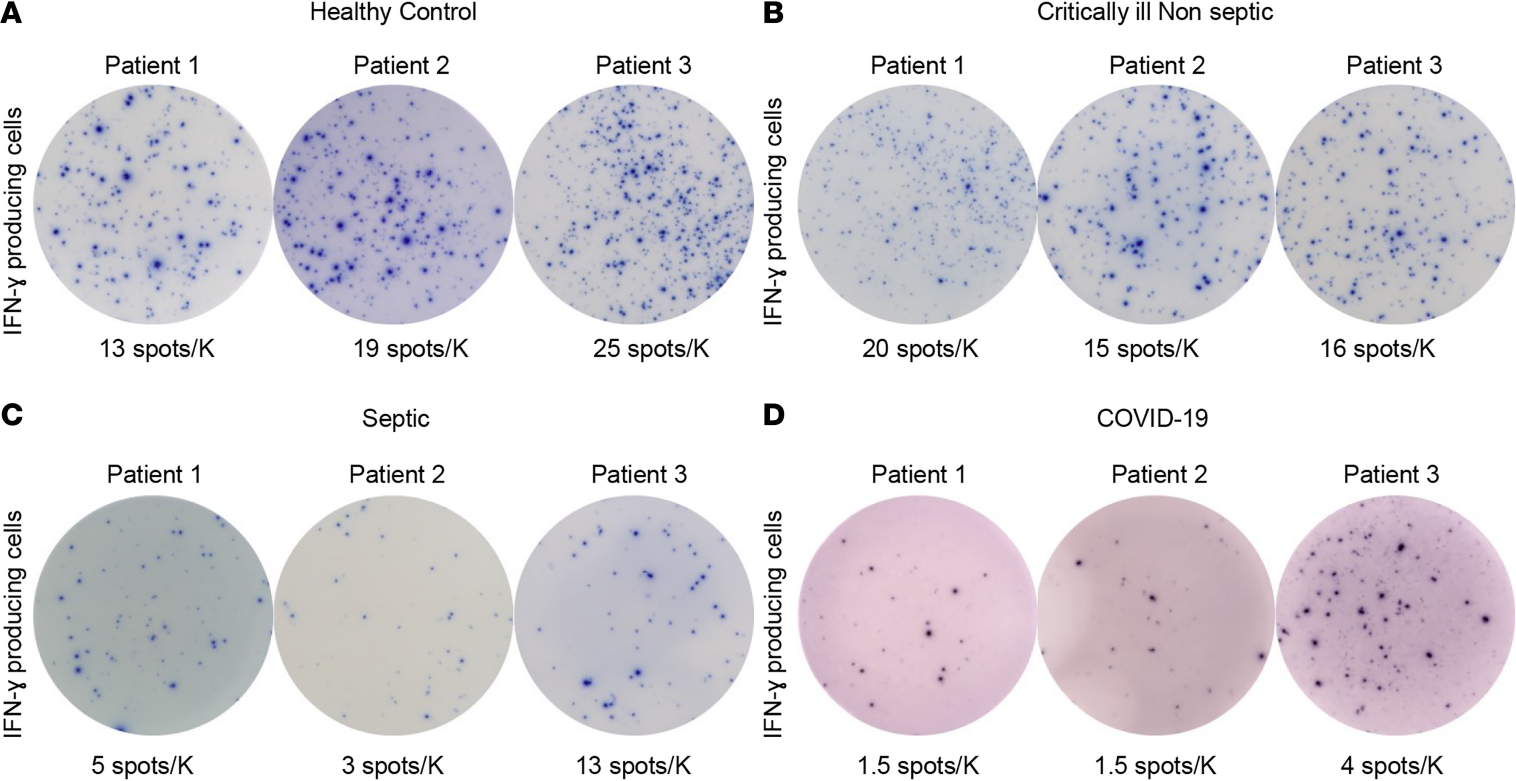
Adaptive immune suppression in COVID-19 patients. Representative ELISpot photomicrographs displaying IFN-ɣ production following overnight stimulation with anti-CD3/anti-CD28 antibodies for (**A**) healthy volunteers, (**B**) CINS patients, and (**C**) septic non–COVID-19 patients. (**D**) Three representative COVID-19–positive samples. Number of spots demonstrates the number of cytokine-producing T cells. Counts are presented as the corrected number of spots per thousand lymphocytes plated as fraction of the 2.5 × 10^4^ PBMCs plated in each well. Note the reduction in IFN-ɣ production in both septic and COVID-19 patients compared with CINS patients. Note also a degree of heterogeneity in IFN-ɣ production in COVID-19 and septic patients. Each photomicrograph was captured with the same magnification, and each image is to scale. ELISpot assays were performed using the PBMC fraction from freshly drawn whole blood. Each condition was run in duplicate for control samples and triplicate for COVID-19 samples.

**Figure 4:**
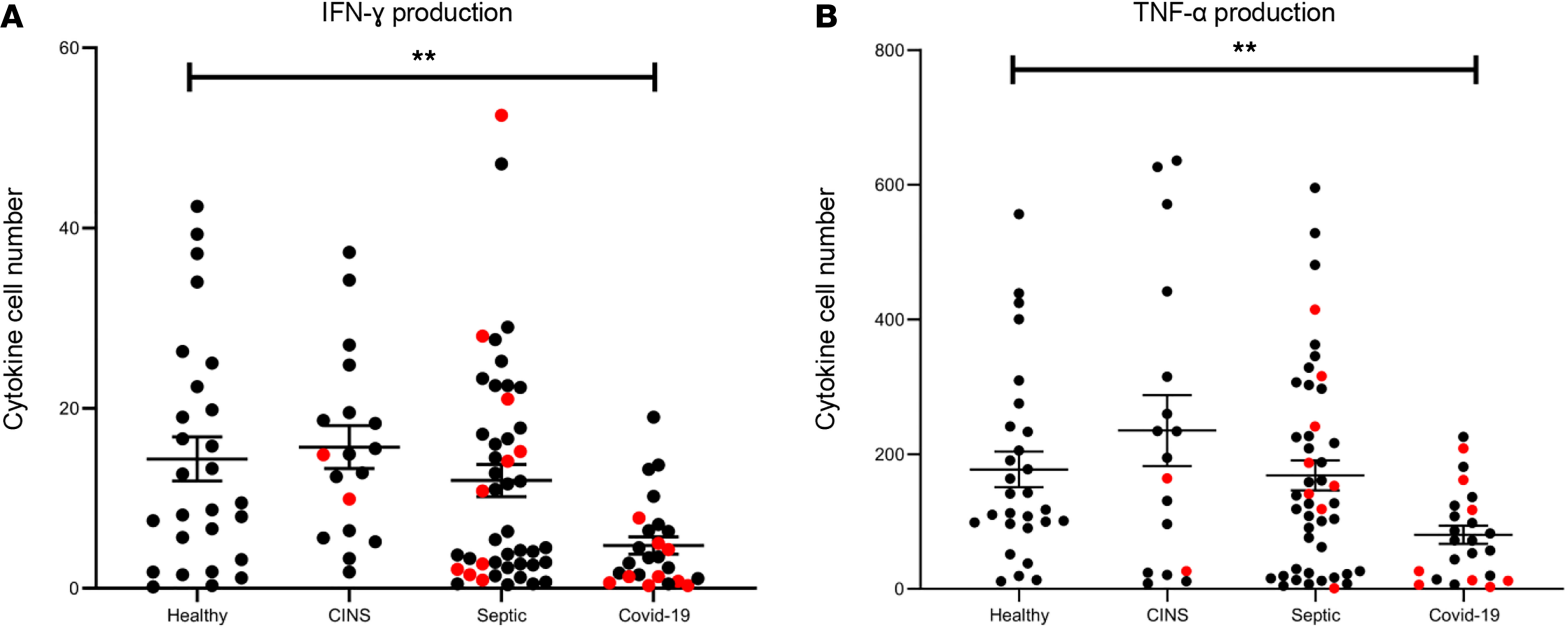
Functional immune cytokine production measured by ELISpot in COVID-19, CINS, and septic patients and healthy volunteers. Comparison graphs for ex vivo cytokine production using ELISpot, comparing healthy volunteers and CINS, septic, and COVID-19 patients. (**A**) Number of spots per 1000 lymphocytes plated following overnight culture stimulated with anti-CD3/anti-CD28 for IFN-ɣ samples. (**B**) Number of spots per 1000 myeloid cells plated, stimulated with LPS for TNF-α production. Each dot represents an individual patient. Red dots represent nonsurvivors. Horizontal bars represent mean ± SEM. Healthy *n* = 27 for IFN-ɣ, 28 for TNF-α; CINS *n* = 18; septic *n* = 46; COVID-19 *n* = 25 for IFN-ɣ, 24 for TNF-α. ANOVA comparing all groups for IFN-γ production showed that there was a difference between COVID-19 and the other groups (*P* = 0.003); and for TNF-α groups there was a statistically significant difference as well (*P* = 0.009). **P < 0.01.

In order to determine the presence and magnitude of functional immunosuppression during COVID-19 infection, we quantitated IFN-ɣ– and TNF-α–producing cells in overnight cell culture in isolated PBMCs by ELISpot analysis after admission. PBMCs were stimulated and incubated overnight with anti-CD3/anti-CD28 to activate T cells, and IFN-ɣ–producing cells were quantified. Data are expressed as positive secreting cells per thousand lymphocytes plated. Representative ELISpot figures for IFN-ɣ–producing cells of representative COVID-19, septic, and CINS patients and healthy volunteers are shown in Figure 3. Quantitatively, the number of cells producing IFN-ɣ in patients with COVID-19 infection was significantly reduced compared with those in all the other groups (*P* = 0.004). Stimulated healthy controls had nearly 3-fold more IFN-ɣ–producing cells than COVID-19 patients (mean 14.4 ± 2.5 vs. 4.8 ± 1). CINS patients had 3-fold-greater levels of stimulated IFN-ɣ production than COVID-19 patients (mean 15.7 ± 2 vs. 4.8 ± 1) Additionally, the mean number of IFN-ɣ–producing cells in septic patients was 2-fold greater than in COVID-19 patients (mean 12 ± 2 vs. 4.8 ± 1) (Figure 4A and Supplemental Figure 1).

### COVID-19 induces profound suppression of monocyte TNF-α.

**Figure 5:**
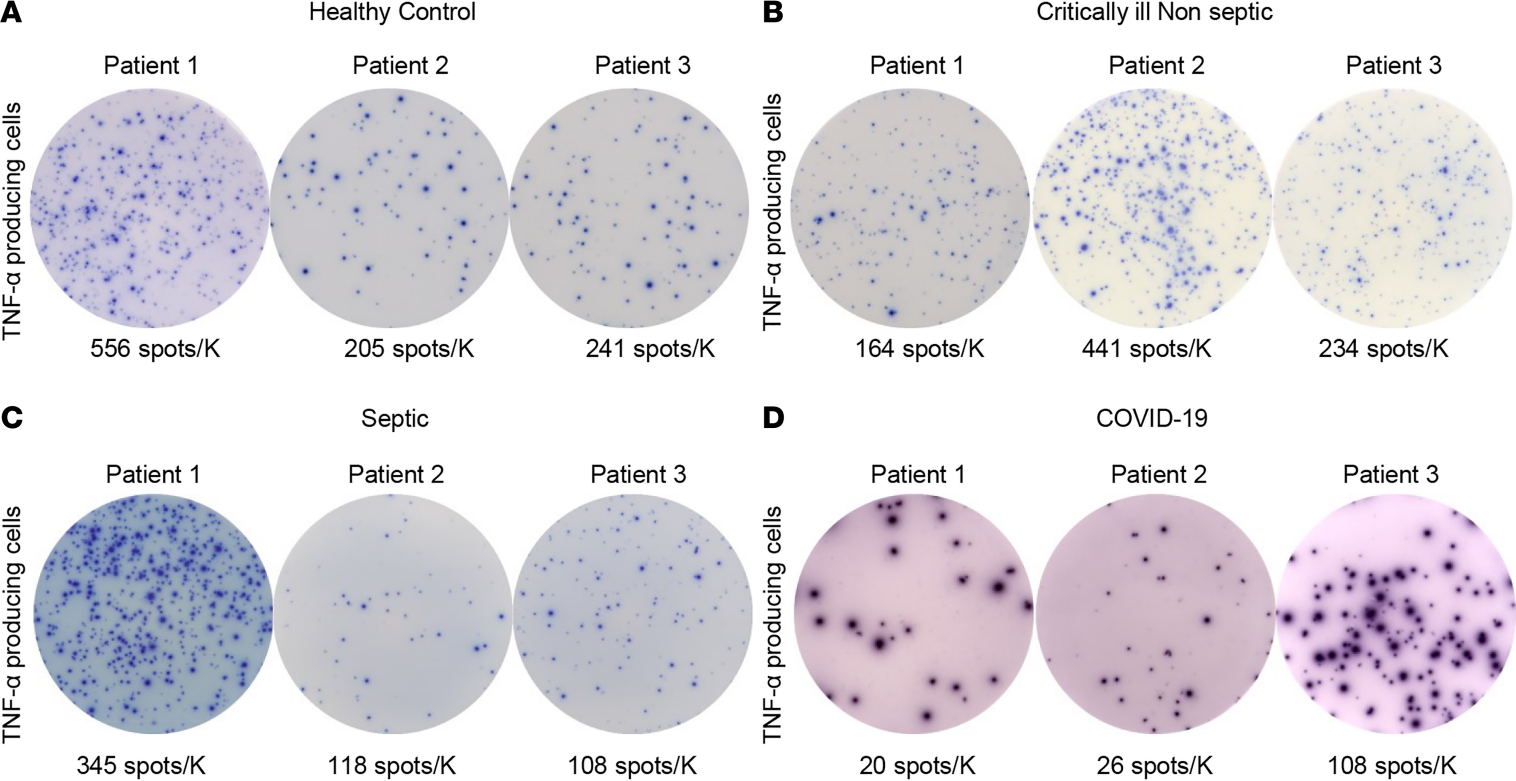
Suppressed innate immune TNF-α response in COVID-19. Representative ELISpot photomicrographs displaying baseline innate immune (monocyte) function with LPS-stimulated TNF-α production in PBMCs. Comparison between different donor types, including (**A**) healthy control volunteers and (**B**) CINS, (**C**) septic, and (**D**) COVID-19 patients. Number of spots demonstrates the number of cytokine-producing monocytes, and counts are presented as corrected number of spots per thousand monocytes plated as fraction of the 2.5 × 10^3^ PBMCs plated in each well. COVID-19 patients had suppressed TNF-α production when compared with controls. Each photomicrograph was captured with the same magnification, and each image is to scale. ELISpot assays were performed using the PBMC fraction from freshly drawn whole blood. Each condition was run in duplicate for control samples and triplicate for COVID-19 samples.

PBMCs were also stimulated overnight with LPS to activate monocytes, and the numbers of TNF-α–producing cells were determined for COVID-19, septic, and CINS patients. Data for TNF-α cytokine–producing cells are expressed as secreting cells per 1000 myeloid cells plated. Representative ELISpot figures for the mean number of TNF-α–producing cells of 3 different COVID-19, septic, and CINS patients and healthy controls are shown in Figure 5.

Importantly, there was considerable patient heterogeneity in TNF-α production as determined by ELISpot assay. A subset of COVID-19 patients had LPS-stimulated TNF-α production that was comparable to that occurring in other critically ill patients, while a large number of COVID-19 patients had reduced production (Figure 4B). None of the COVID-19 patients had increased TNF-α production in response to LPS stimulation.

Quantitatively, the number of cells producing TNF-α was reduced 3-fold and 2-fold in with COVID-19 compared with CINS and septic patients, respectively (*P* = 0.009; mean CINS: 272 ± 64; septic: 168 ± 22; COVID-19: 80 ± 14). Compared with healthy volunteers, stimulated PBMCs from COVID-19 patients had half as many TNF-α–producing cells (healthy, 177.5 ± 27) (Figure 4B and Supplemental Figure 2).

Both innate and adaptive immune cells from COVID-19 patients who experienced mortality within 30 days of ICU admission were among the most phenotypically suppressed samples. COVID-19 nonsurvivors had quantitatively low ELISpot IFN-ɣ and TNF-α production, although the difference was not statistically significant (Figure 4, red dots).

### Sustained immune suppression over time in patients with COVID-19.

**Figure 6:**
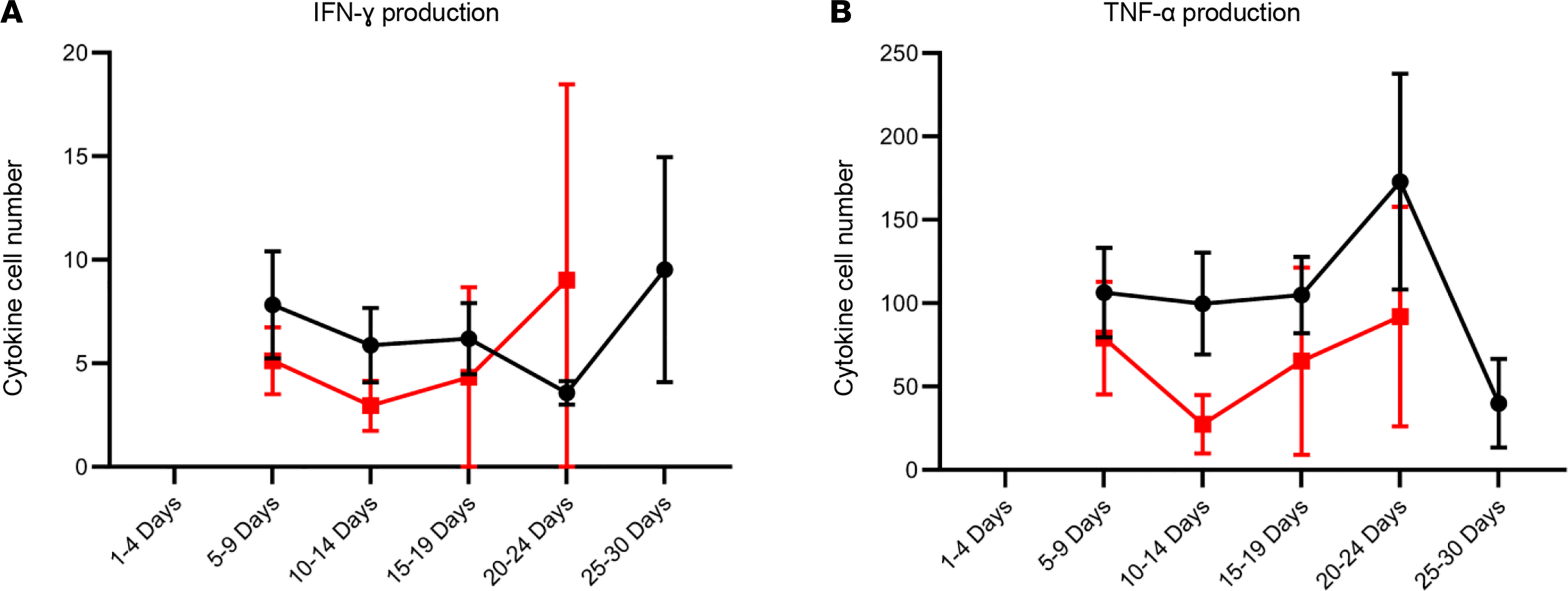
Number of cytokine-producing cells in COVID-19 patients serially over time. Time course analysis of ELISpot results comparing (**A**) IFN-ɣ and (**B**) TNF-α production in COVID-19 survivors versus nonsurvivors (red) from onset of illness throughout ICU admission. There was no statistical significance between survivors and nonsurvivors using a modified *t* test. Day of illness data were collected via chart review. Horizontal bars represent mean ± SEM. For each time point, there are the following number of samples: IFN-ɣ survivors: 0, 8, 10, 10, 8, 4; IFN-ɣ nonsurvivors: 0, 4, 7, 3, 3, 0; TNF-α survivors: 0, 8, 7, 9, 6, 3; TNF-α nonsurvivors: 0, 5, 5, 4, 3, 0.

COVID-19 patients were followed over time with serial ELISpot assays, and the mean number of IFN-ɣ– and TNF-α–producing cells remained suppressed and did not increase over the time course of disease (IFN-ɣ *P* = 0.54, TNF-α *P* = 0.42) (Figure 6). Although nonsurvivors maintained lower numbers of IFN-ɣ– and TNF-α–producing cells than survivors this did not reach statistical significance.

### Profound depletion of CD4^+^ and CD8^+^ T cells in COVID-19.

**Figure 7:**
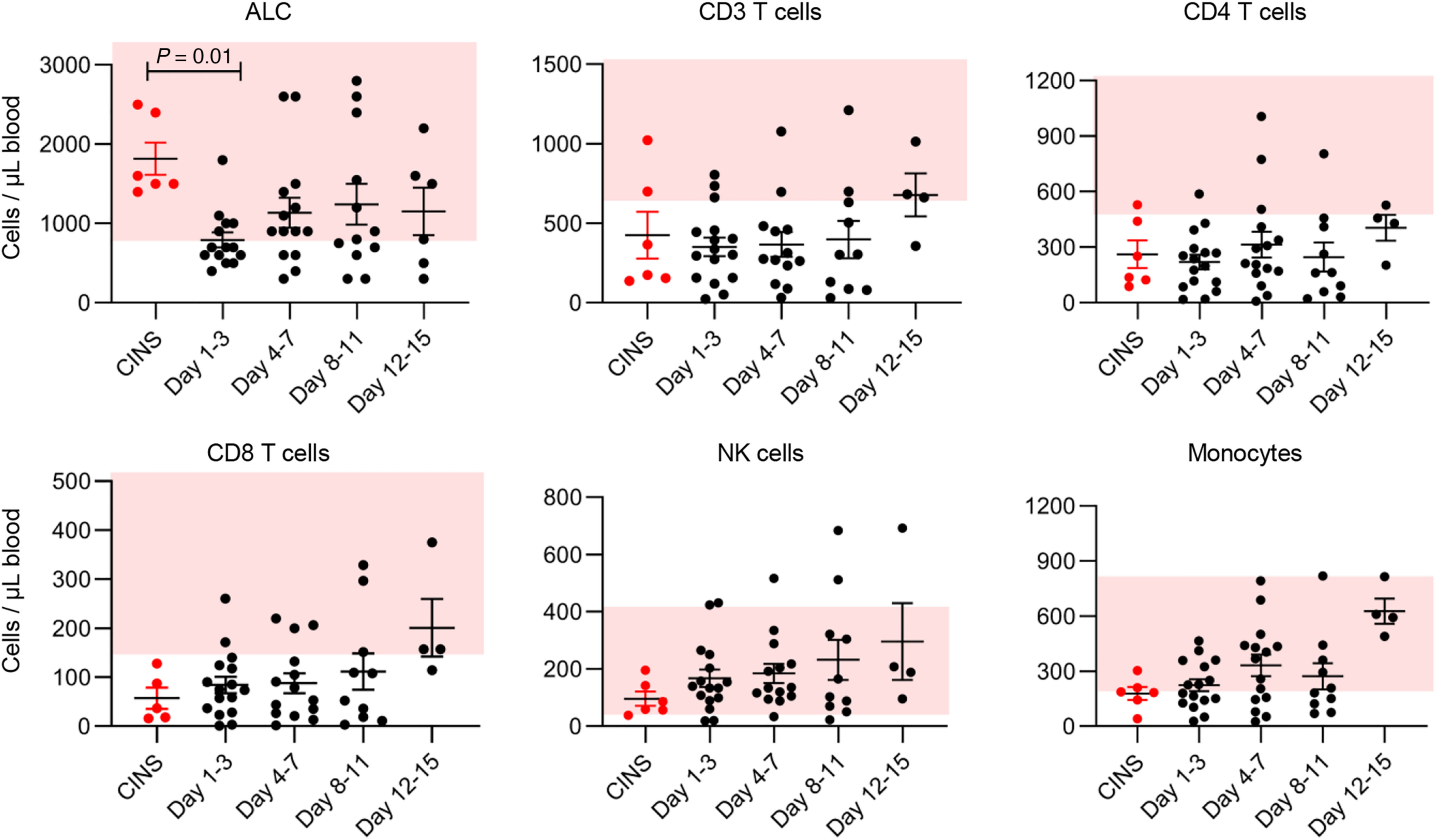
COVID-19 induces profound depletion of circulating immune effector cells. Absolute numbers of various white blood cell types (displayed as cells/μL) were determined in COVID-19–positive and CINS patients (red dots). ALC was determined by Barnes-Jewish Hospital Clinical Laboratory as part of patient clinical laboratory tests. CD3^+^ T, CD4^+^ T, CD8^+^ T, and NK cell and monocyte quantification was performed using flow cytometry as described in Methods. Pink shading represents normal reference values for healthy individuals at Barnes-Jewish Hospital Laboratories. Analysis by ANOVA with Dunnett’s multiple comparison tests showed a significant decrease in ALC from CINS to COVID-19 days 1–3; *P* = 0.01. ALC for CINS *n* = 6; ALC for COVID-19 days 1–3 *n* = 15, days 4–7 *n* = 14, days 8–11 *n* = 12, days 12–15 *n* = 6. Cell counts for CD3^+^, CD4^+^, CD8^+^, and NK cells and monocytes: CINS *n* = 6; COVID-19 days 1–3 *n* = 15, days 4–7 *n* = 14, days 8–11 *n* = 10, days 12–15 *n* = 4.

Flow cytometric analysis of samples was performed in all COVID-19 patients (days 1–3, 4–7, 8–11, and 12–15) and in CINS patients (days 1–3) as previously described (33, 34). ALC was profoundly depressed in COVID-19 patients over the entire duration of the study compared with nonseptic patients (first comparison days 1–3; *P* = 0.01) (Figure 7A). Next, we evaluated absolute CD3^+^, CD4^+^, and CD8^+^ T cell, NK cell, and monocyte numbers (Figure 7, B–F). CD3^+^, CD4^+^, and CD8^+^ T cell numbers were severely depressed compared with those in the normal range (pink shaded area) reported for healthy individuals at the Clinical and Diagnostic CLIA-CLA Laboratories at Barnes-Jewish Hospital (St. Louis, Missouri, USA), and remained suppressed for the duration of the study. Although CINS patient samples were not followed sequentially, their initial values were low, similar to the levels found in COVID-19 patients.

### IL-7 increases T cell IFN-ɣ production in COVID-19.

**Figure 8:**
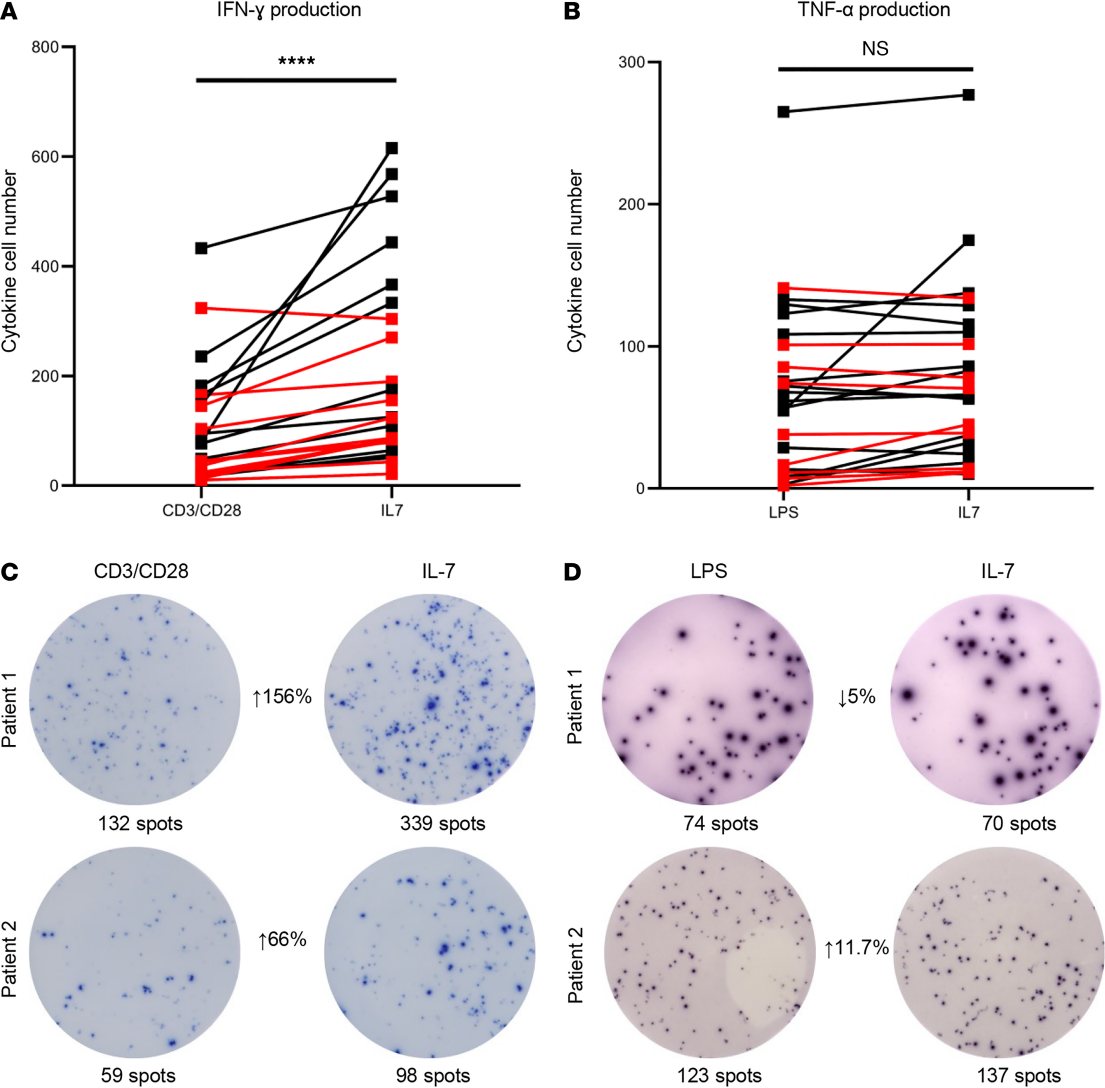
IL-7 restores adaptive immune function in patients with COVID-19. Line plot demonstrating change in the number of cytokine-producing cells using ELISpot between control (anti-CD3/anti-CD28 antibody or LPS) samples and stimulation with IL-7 for IFN-ɣ (**A**) and TNF-α (**B**). Each dot represents and individual patient. Red lines represent values for patients who died. IL-7 caused a significant increase in the number of IFN-ɣ–producing T cells in COVID-19 patients; ****P < 0.0001. IL-7 did not increase monocyte TNF-α production. (**C** and **D**) Representative photomicrographs demonstrating ELISpot change from control sample to IL-7 stimulated for IFN-ɣ and TNF-α. Paired samples were analyzed using a paired Wilcoxon’s rank-sum test. IFN-ɣ *n* = 25, TNF-α *n* = 25.

To test the potential efficacy for IL-7 as an immunoadjuvant therapy to restore COVID-19–induced T cell exhaustion, we cocultured patient-derived PBMCs with IL-7 for ELISpot analysis. The mean number of IFN-ɣ–producing T cells from COVID-19 patients nearly doubled, from 101 ± 21 to 201 ± 36 (*P* < 0.0001), following ex vivo administration of IL-7 (Figure 8A). Although there was an increase in LPS-induced TNF-α–producing cells in some samples, and a mean increase of 101% overall after IL-7 coincubation, these changes were not statistically significant (Figure 8B). The effect of IL-7 to increase the number of IFN-ɣ–producing T cells was also observed in septic and CINS patients (Supplemental Figures 3 and 4).

## Discussion

Currently, the prevailing paradigm that guides the therapeutic approach to COVID-19 is that patients are dying from the effects of cytokine storm–mediated inflammation with resultant lung and other organ injury (6, 7, 40–43). Based on this theory of unbridled inflammation, COVID-19 patients are currently being treated with a variety of drugs that block proinflammatory cytokines or inhibit the inflammatory signaling cascade. The results from the present study strongly suggest that the primary endotype of COVID-19 is one of immunosuppression rather than hyperinflammation. Therefore, the approach of broadly inhibiting the host inflammatory response may be misguided, and may actually worsen clinical trajectories in some COVID-19 patients due to further impairment of an already compromised host protective immune response. Circulating cytokines in COVID-19 patients, at least early in their clinical course, did not show widespread elevation. Most COVID-19 patients had either no elevation or only mild increases in the major proinflammatory cytokines including TNF-α, IL-1α, IL-1β, IFN-ɣ, etc. (Table 2). There were modest elevations in plasma IL-6 in COVID-19 patients, with only 6 patients reaching IL-6 concentrations greater than 1000 pg/μL, as typically seen during overwhelming bacterial sepsis or cytokine release syndrome (44, 45). There were 2 additional COVID-19 patients who had IL-6 levels close to 1000 pg/mL as well as 4 patients whose IL-6 levels were above the level of detection for the assay. Of the aforementioned patients, sustained elevation of IL-6 was detected in some, while others had variable fluctuations in IL-6 levels over time. In addition to macrophages, IL-6 can be made by many different types of cells, including pulmonary epithelial cells, infected with coronaviruses (1, 46). Thus, the increase in IL-6 and IL-8 concentrations that occurs in COVID-19 infection may be a reflection of virus-induced epithelial cell production or cell injury, rather than evidence of a systemic hyperinflammatory response.

In addition, there was no evidence of exaggerated TNF-α production in response to ex vivo LPS stimulation of PBMCs when compared with septic and CINS patients, nor did the patients have elevated plasma TNF-α levels. Rather, the findings show a predominant endotype of immunosuppression, manifesting as both a profound and sustained loss of CD4^+^ and CD8^+^ T cells, as well as a reduced responsiveness of the remaining lymphocytes to T cell receptor activation. These cells and their responsiveness are essential to containing and eliminating viral pathogens (47). The key finding in the present study is that there is not only a loss in the number of immune cells, but also an accompanying critical defect in the responsiveness of surviving lymphocytes and monocytes.

A potentially novel aspect of the present study is the use of ELISpot assays performed on freshly obtained blood samples to evaluate individual immune cell responsiveness to agonists. The ELISpot method provides an improved readout of cell function with enhanced sensitivity and increased dynamic range compared with flow cytometric techniques (15, 38). The ELISpot assay showed that when compared with CINS patients, stimulated PBMCs from COVID-19 patients will only activate approximately half the number of IFN-ɣ–producing lymphocytes (*P* < 0.0001). Similar declines were seen in LPS-stimulated TNF-α production by monocytes from COVID-19 patients. Interestingly, COVID-19 patients who died appeared to have the most profound suppression of TNF-α and IFN-ɣ production (Figure 4), and the immune suppression was sustained through at least the first 3 weeks after ICU admission (Figure 6).

Both clinical and pathological findings suggest that immunosuppression is a critical pathophysiologic phenomenon of COVID-19. Zhou et al. reported that 50% of COVID-19 patients who die develop secondary hospital-acquired infections (48). Autopsy studies of COVID-19 patients demonstrate inclusion bodies, pathologic findings consistent with viral persistence within cells present in lung, kidney, and other organs (23, 49, 50). A recent autopsy investigation of 12 patients who died of COVID-19 showed that 11 of the patients had up to 500,000 viral copies/1 × 10^6^ RPPH1 copies in lung tissue by SARS-CoV2–specific RT-qPCR (51). Ten of the 12 patients had superimposed bronchopneumonia with both focal and diffuse distribution. Collectively, these studies suggest that there is an inability of the host to mount an adequate immune defense, leading to viral dissemination and organ injury and rendering the patient more susceptible to subsequent hospital-acquired infections.

One important implication of the massive depletion and impaired function of lymphocytes is that immune adjuvants that enhance host immunity should be strongly considered as potential therapeutic interventions in patients with COVID-19. Decades of mechanistic immunologic studies have invariably demonstrated that an intact T cell–mediated adaptive immune response is required for eliminating and suppressing viral infections (52). Support for this potential immune therapeutic approach is provided by studies showing that checkpoint inhibitors and common γ-chain cytokines, which stimulate CD4^+^ and CD8^+^ T cells, have been effective in a number of serious viral infections, including hepatitis C, JC virus–induced progressive multifocal leukoencephalopathy, and HIV (47, 53). Several of these agents (NKG2D-ACE2 CAR-NK cells, anti–PD-1, IL-7) are either in active clinical trials or in the planning stages for COVID-19 (NCT04324996, NCT04356508, NCT04379076, respectively).

Of particular relevance regarding potential immune adjuvant therapy for COVID-19 are the ELISpot results showing that ex vivo IL-7 increased IFN-ɣ production of stimulated T cells nearly 2-fold (Figure 8). A clinical trial of IL-7 in patients with sepsis showed that IL-7 was well tolerated, reversed sepsis-induced lymphopenia, and increased CD4^+^ and CD8^+^ T cells by 2- to 3-fold (54).

Another important implication of the present study is that ELISpot may be used to phenotype COVID-19 patients to determine appropriate immunomodulatory drug therapies. Results of the ELISpot analysis showed that some COVID-19 patients displayed ex vivo cytokine production, comparable to results from CINS patients (Figure 4). Therefore, use of immunostimulant therapies to restore protective immunity in these patients might not be indicated. Conversely, COVID-19 patients with severe reductions in T cell or monocyte cytokine production might benefit from agents to boost their host immunity. We would expect that the ELISpot assay could be used clinically to evaluate the progression of immune dysfunction and to evaluate the effect of different immune therapies to restore innate and adaptive immunity in an immunosuppressed patient.

### Limitations.

There are several limitations to the present study. Most of the COVID-19 patients had symptoms of infection several days prior to hospitalization (Figure 1). Although an early and excessive hyperinflammatory phase may have already occurred prior to hospitalization, we deem this unlikely, because significant systemic inflammatory reactions typically induce hypotension and dyspnea that would have led patients to seek immediate care. A second limitation to this study is that it does not exclude a subset of COVID-19 patients who do have cytokine storm–mediated hyperinflammation with accompanying lung and organ injury. Thus, anti-cytokine therapy or drugs to negatively modulate the inflammatory response may be beneficial in this subset of patients. However, the present results show that a markedly immunosuppressive phenotype predominates in COVID-19 patients. The ongoing clinical trials of anti-cytokine agents and immunosuppressive therapies will likely resolve whether COVID-19 patients actually have damaging hyperinflammatory responses. Ultimately, in order to eradicate the virus, patients need a competent and active immune system, and research should focus on such therapies to restore this vital function.

Finally, the present results, which are based on blood measurements, do not exclude the possibility that damaging inflammation occurs locally within the lung and other organs that is not detected by levels of circulating cytokines or ELISpot analysis of PBMCs. Direct examination of samples obtained by bronchoalveolar lavage would help address this issue of potential compartmentalized responses to COVID-19 infection.

### Conclusions.

We conclude that the major immunologic abnormality in COVID-19 is a profound defect in host immunity and not hypercytokinemia-induced organ injury. The defect in host immunity includes both a profound depletion in the number of effector immune cells and severe functional defects in T cell and monocyte function. Based on these findings, immunoadjuvant therapies to enhance host immunity should be considered. Evaluating patient innate and adaptive immunity using functional assays such as ELISpot may be useful in guiding immunomodulatory therapies. IL-7 reverses T cell exhaustion in COVID-19 and should be considered as a potential therapy in this highly lethal disorder.

## Methods

### Study design

This was a prospective observational cohort study among patients with COVID-19 in a mixed medical and surgical ICU between March 2020 and May 2020 at Missouri Baptist Medical Center and Barnes-Jewish Hospital. Additionally, samples obtained previously (in 2018–2020) from sepsis or CINS patients were used for comparison.

Patient demographic data, including clinical course, relevant laboratory testing, onset of symptoms prior to admission to the hospital, morbidity, mortality, and medical management data were collected and deidentified. Complete blood counts were recorded at the time closest to blood sampling for immune functional testing. For the COVID-19 patients, the first study blood sample was obtained within the first 24 hours from clinical deterioration (endotracheal intubation) after admission to the ICU in order to try to capture the early hyperinflammatory phase of infection. COVID-19 patients had 2 blood draws weekly, for a maximum of 4 blood draws, and septic patients had the option for a redraw at 1 week if the patient remained in the ICU.

### Inclusion criteria

We included hospitalized patients, aged 18 years or older, who were COVID-19 positive via either nasopharyngeal- or tracheal aspirate-derived SARS–CoV-2 RNA using an FDA-approved clinical PCR test. COVID-19 testing results were available from 6 to 30 hours after hospital admission. For inclusion in the study, patients with sepsis were defined as previously described (54), including the presence of 2 or more criteria for systemic inflammatory response syndrome (SIRS), 2 or greater point increase in SOFA score, and clinically or microbiologically suspected infection. CINS patients included patients admitted to the medical or surgical ICU following major surgical procedures or major traumatic injury or with noninfectious causes of organ failure, requiring intensive care management and not showing evidence of infection. Healthy control participants had no ongoing infections or autoimmune disease, and no past history of cancer or solid organ transplant.

### Exclusion criteria

No screened patients were excluded from the COVID-19 cohort. For the critically ill groups, to minimize confounding effects of immunosuppressive medications or underlying immunologic disease, patients with the following criteria were excluded: (i) patients with active cancer and/or undergoing chemotherapy or radiation treatment within the past 6 weeks; (ii) HIV; (iii) known history of acute or chronic lymphocytic leukemia; (iv) pregnancy; (v) organ or bone marrow transplantation; (vi) use of current high-dose corticosteroid regimens that were greater than or equivalent to 300 mg/d hydrocortisone or other immunosuppressive medications; (vii) current use of immune-modifying biological agents including inhibitors of TNF-α or other cytokines, viral hepatitis, or systemic autoimmune diseases; and (viii) participation in another interventional trial within the past 4 weeks

### Specific laboratory studies

#### Plasma cytokine measurements.

Cytokine quantitation was performed on plasma obtained from patients (frozen at –80°C prior to use), and subsequently analyzed using a human MagPix multiplex cytokine panel (Invitrogen) and on a Luminex FLEXMAP 3D instrument according to the manufacturer’s instructions.

#### ELISpot quantitation of IFN-ɣ and TNF-α production.

Quantitation of IFN-ɣ– and TNF-α–producing cells was performed on isolated PBMCs by ELISpot analysis, as per the manufacturer’s instruction (Cellular Technologies Limited [CTL] Immunospot, R&D Systems) and as previously described (38, 39). Patient PBMCs were harvested from whole blood via Ficoll-Paque, counted using the Vi-Cell counter from Beckman Coulter, and incubated overnight plated in 96 well ELISpot culture plates with CLT media or RPMI 1640 media (Sigma-Aldrich) supplemented with human AB serum, nonessential amino acids, penicillin/streptomycin, and l-glutamine. Septic and CINS patient samples were plated in duplicate, and COVID-19 subject samples were plated in triplicate; these results were averaged for each patient sample. ELISpot plates were used for capture of both IFN-ɣ and TNF-α. For R&D kits, when used, capture antibody was prepared and placed in wells as per the manufacturer’s recommendations. CTL kits came with capture antibody precoated. Cells plated in IFN-ɣ wells were plated at a standardized density of 2.5 × 10^4^ and 5 × 10^4^ PBMCs per well and stimulated with anti-CD3 (clone HIT3a; BioLegend) and anti-CD28 (clone CD28.2; BioLegend) antibodies at 1 μg/mL. Cells plated in TNF-α wells were plated at a standardized density of 2.5 × 10^3^ and 5 × 10^3^ PBMCs per well, and 5 × 10^3^ were stimulated with 100 ng/ml LPS (from *Salmonella abortus equi* S-form, ALX-581-009, Enzo Life Sciences). Anti-CD3 with anti-CD28 or LPS was used as stimulant to evaluate the baseline function of T cells and monocytes, respectively, to assess ability to produce and secrete IFN-ɣ or TNF-α. ELISpot plates were made by Merck Millipore and obtained through Thermo Fisher Scientific (M8IPS4510). Spots were detected using a colorimetric reagent kit (Strep-AP and BCIP-NBT, R&D Systems, SEL002). Following development, images were captured and analyzed on CTL ImmunoSpot 7.0 plate reader and software.

The immunoadjuvant, IL-7, was obtained from R&D Systems (catalog 207-IL-200). Additional ELISpot wells were prepared as mentioned above with the addition of IL-7 at a final concentration of 50 ng/mL.

### Flow cytometry

Flow cytometric analysis of samples was performed as previously described (39, 55). Briefly, whole blood or PBMCs were stained for 30 minutes at room temperature, and red blood cells lysed (in the case of whole blood) using Red Blood Cell Lysis Buffer (BioLegend). Samples were acquired on an Attune NxT cytometer (Thermo Fisher Scientific) and data analyzed using FlowJo 10.6.2 (BD Biosciences). Absolute cell counts were ascertained by use of counting beads in LUCID DURAclone staining tubes (Beckman Coulter). The gating strategy used is shown in Supplemental Figures 5 and 6.

The following antibodies (clones) were used in this work: CD3 (HIT3a)–FITC, CD14 (M5E2)–PerCP/Cy5.5, CD4 (RPA-T4)–APC/Cy7, CD8 (SK1)–APC, CD56 (5.1H11)–BV711, CD14 (M5E2)–BV650 (BioLegend), CD3 (UCHT1)–FITC, CD4 (13b8.2)–PacificBlue, and CD8 (B9.11)–KromeOrange (Beckman Coulter).

### Statistics

All statistical analyses were performed using GraphPad Prism version 8.4 and SPSS Statistics version 25 (IBM). Mean percentage change in spot number was calculated by dividing the difference between the control and treatment sample by the value of the control. Statistical analysis of ELISpot data comparing unstimulated results with stimulated results was performed using paired analysis with nonparametric Wilcoxon’s signed-rank test. In this test, each patient sample is compared with its own unstimulated control, and these changes are compared for the entire group to determine statistical significance. Mann-Whitney *U* tests were used to compare the mean ELISpot results between different cohorts under similar stimulations. Comparisons of differences in continuous variables within a group (isotype control vs. treatments) were done using paired Student’s *t* tests, 1-way ANOVA, and multivariate analysis. *P* values less than 0.05 were considered significant.

ELISpot results were corrected for number of cells plated in the following method: The number of spots determined using the CTL ELISpot analyzer represents the number of cells secreting the relevant cytokine. PBMC IFN-ɣ spots were corrected as the number of spots per lymphocyte percentage in the PBMC fraction based on flow cytometry data. PBMC TNF-α spots were corrected as the number of spots per myeloid cell percentage in the PBMC fraction. For COVID-19 samples, flow cytometry was performed on the PBMC fraction, and neutrophil contamination was included in the correction fraction. Spot number for IFN-ɣ and TNF-α was reported per thousand cells plated. For samples that did not have flow cytometry data available, complete blood count with differential was used.

### Study approval

Blood sampling and data collection and analysis were approved by the Institutional Review Boards at Washington University School of Medicine in St. Louis and at Missouri Baptist Hospital, St. Louis, Missouri, USA (protocols WUSTL 201211101, WUSTL 201603006, WUSTL 202003085, and 201808049; and MOBAP 1132). Written informed consent was obtained from healthy control participants and patients or their legally authorized representatives.

## Author contributions

KER, SCB, MM, LLM, AHE, PAM, and RSH designed the study, supervised experiments, and analyzed and interpreted the data; KER, SCB, LLM, MM, AHW, JU, and RSH wrote/edited the manuscript; MM, TMB, JU, and AHW performed all statistical analyses; MM, PAM, DJY, AHW, JU, DAM, and DFO performed relevant experiments; JB and TMB provided study coordination; DAS, RSM, NJA, JPB, AMD, CCC, and IRT provided analysis and edited the manuscript; JU, MM, TMB, and AHW provided figure generation. All authors reviewed and approved the final version of the manuscript.

## Supplementary Material

Supplemental data

## Figures and Tables

**Table 2:**
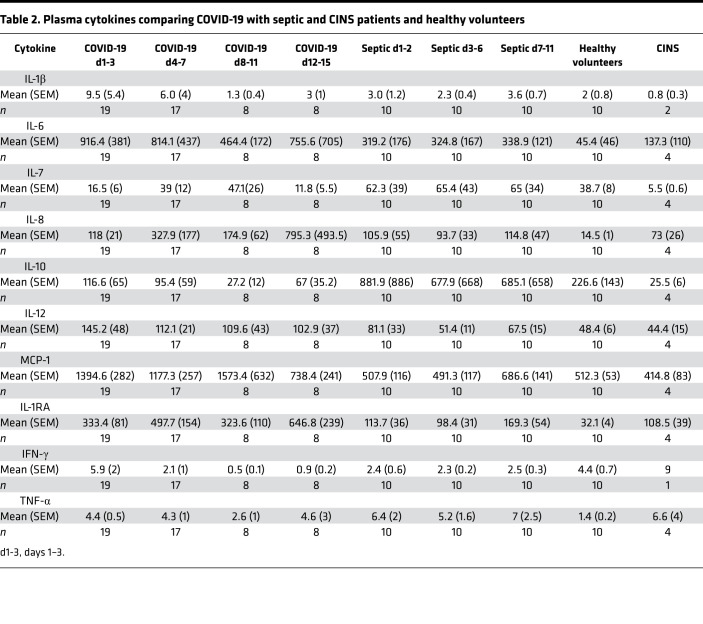
Plasma cytokines comparing COVID-19 with septic and CINS patients and healthy volunteers

**Table 1:**
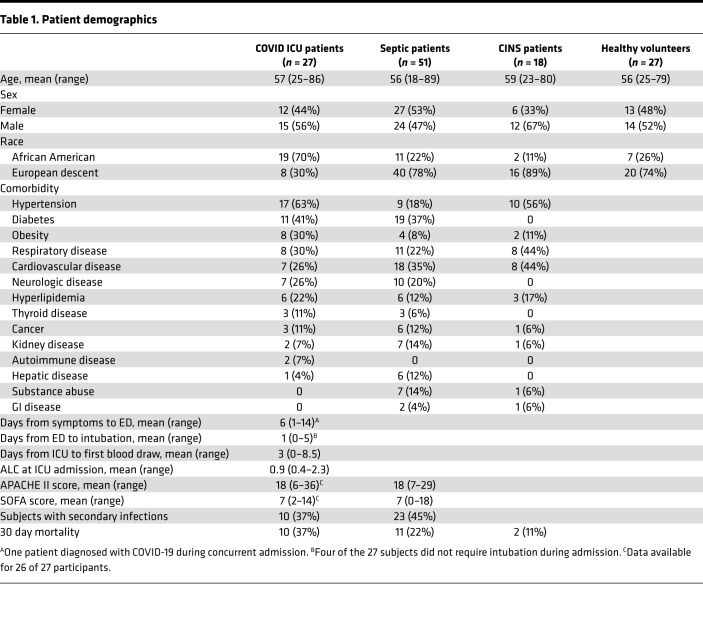
Patient demographics

## Version 1 07/20/2020

In-Press Preview

## Version 2 09/03/2020

Electronic publication
