# Supplementary material for: Severe immunosuppression and not a cytokine storm characterizes COVID-19 infections
Source: JCI Insight. 2020 Sep 3;5(17):e140329. doi: 10.1172/jci.insight.140329 (PMC7526441; doi:10.1172/jci.insight.140329)
Supplement: Supplemental data [file jciinsight-5-140329-s125.pdf]

Supplemental Figure 1:

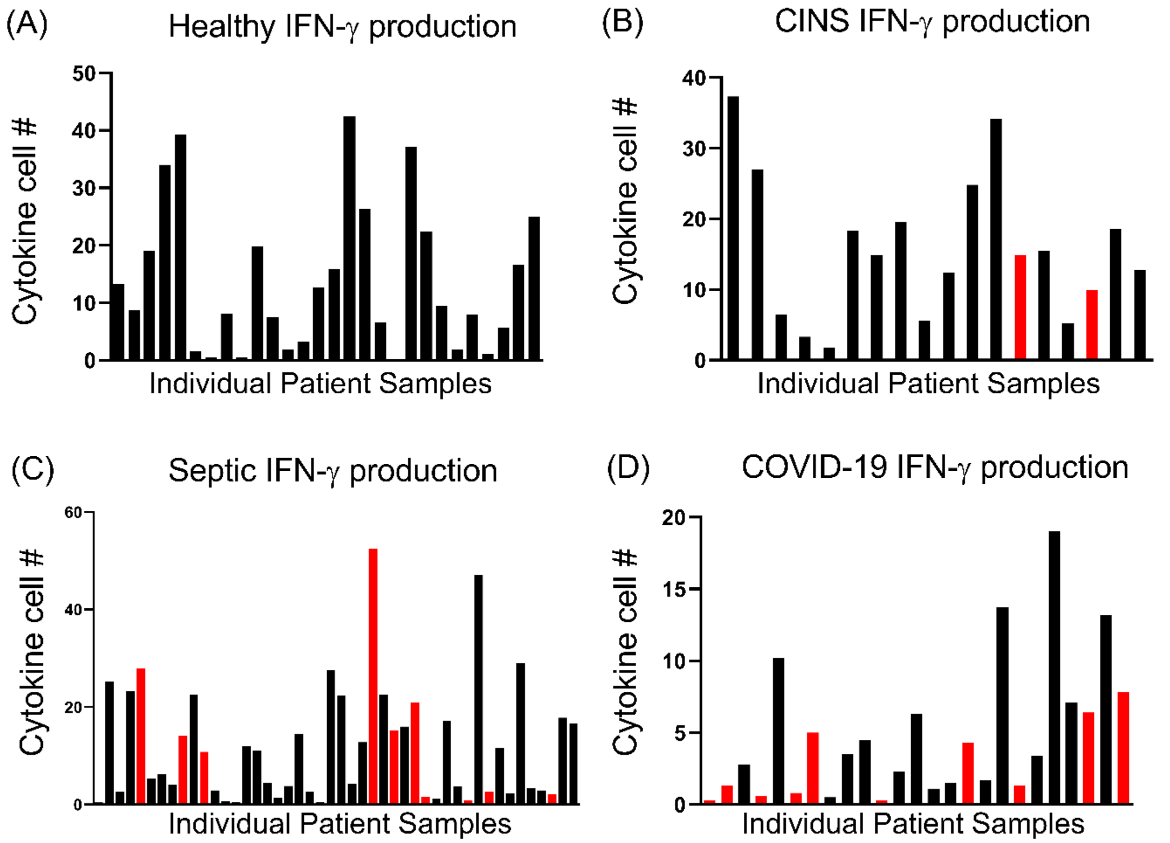

Supplemental Figure 1. Individual patient IFN-ɣ ELISpot production for healthy, CINS, septic and COVID-19

Bar graphs for individual patient immune response, represented as number of IFN-ɣ cytokine producing T cells. (A) Healthy Control (n = 27), (B) Critically ill non-septic (n = 18). (C) Septic patients (n = 46). (D) COVID-19 positive patients (n = 25). Each bar represents an individual patient. Red lines indicate patients who died. ELISpot assays were performed in duplicate for controls and triplicate for COVID-19 patients.

Supplemental Figure 2:

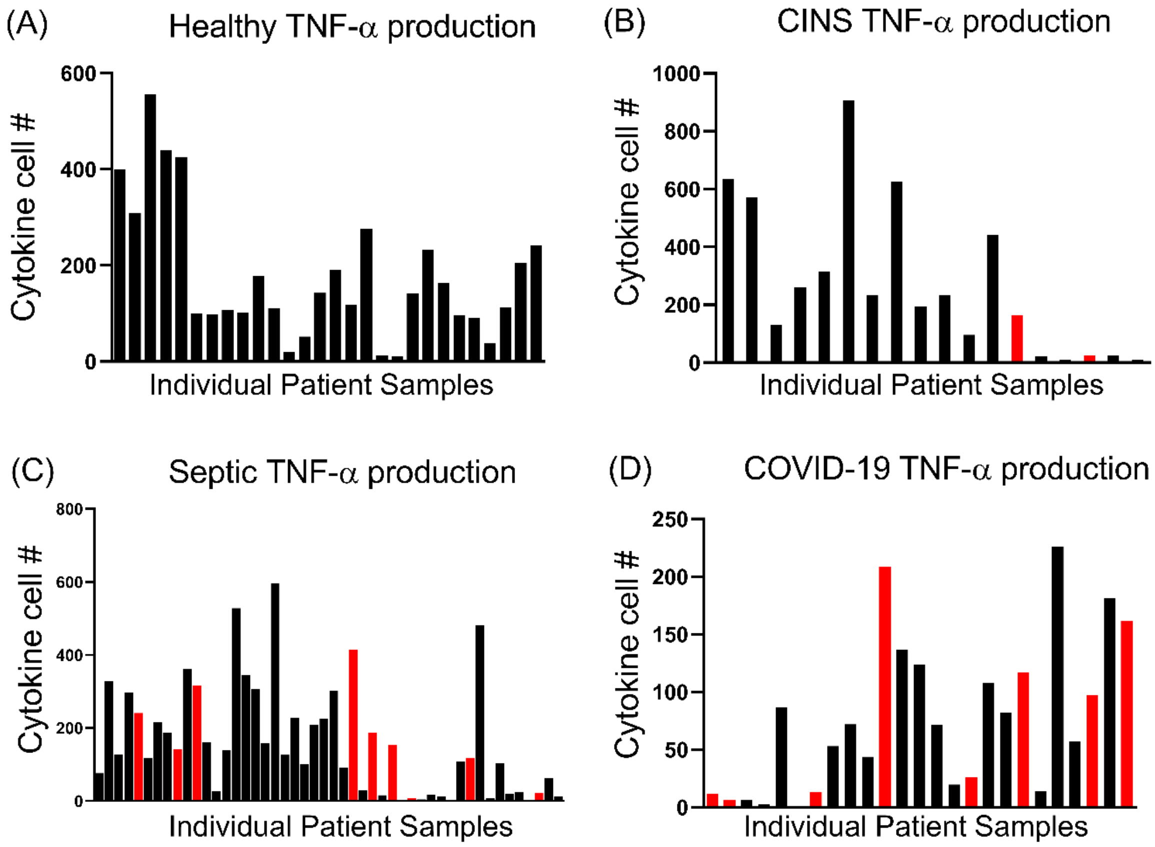

Supplemental Figure 2. Individual patient TNF-α ELISpot production for healthy, CINS, septic and COVID-19

Bar graphs for individual patient immune response, represented as number of TNF-α producing monocytes. (A) Healthy Controls (n = 27), (B) Critically ill non-septic (n = 18). (C) Septic patients (n = 46). (D) COVID-19 positive patients (n = 24). Each bar represents an individual patient. Red lines indicate patients who died. ELISpot assays were performed in duplicate for controls and triplicate for COVID-19 patients.

Supplemental Figure 3:

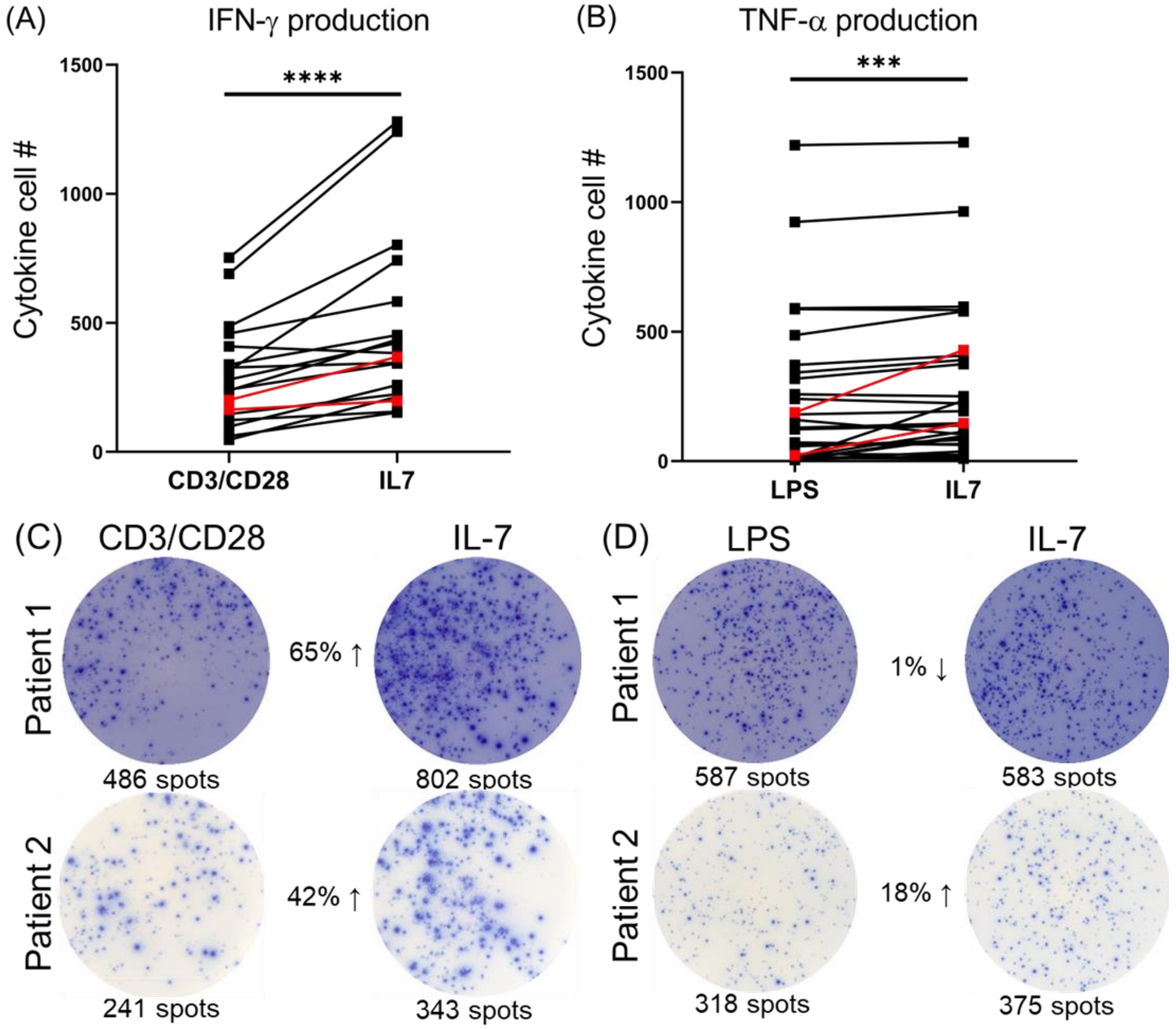

Supplemental Figure 3. IL-7 Effect on the innate and adaptive immune function in critically ill non-septic patients.

Line plot demonstrating change in number of cytokine producing cells using ELISpot between control (anti-CD3/anti-CD28 antibody or LPS) samples and stimulation with IL-7 for IFN-ɣ (A) and TNF-α (B). Panels (C), (D) are representative photomicrographs demonstrating ELISpot change from control sample to IL-7 stimulated for IFN-ɣ and TNF-α. Paired samples were analyzed using a paired rank sum Wilcoxon test. IFN-ɣ n= 25, TNF-α n=25.

Supplemental Figure 4:

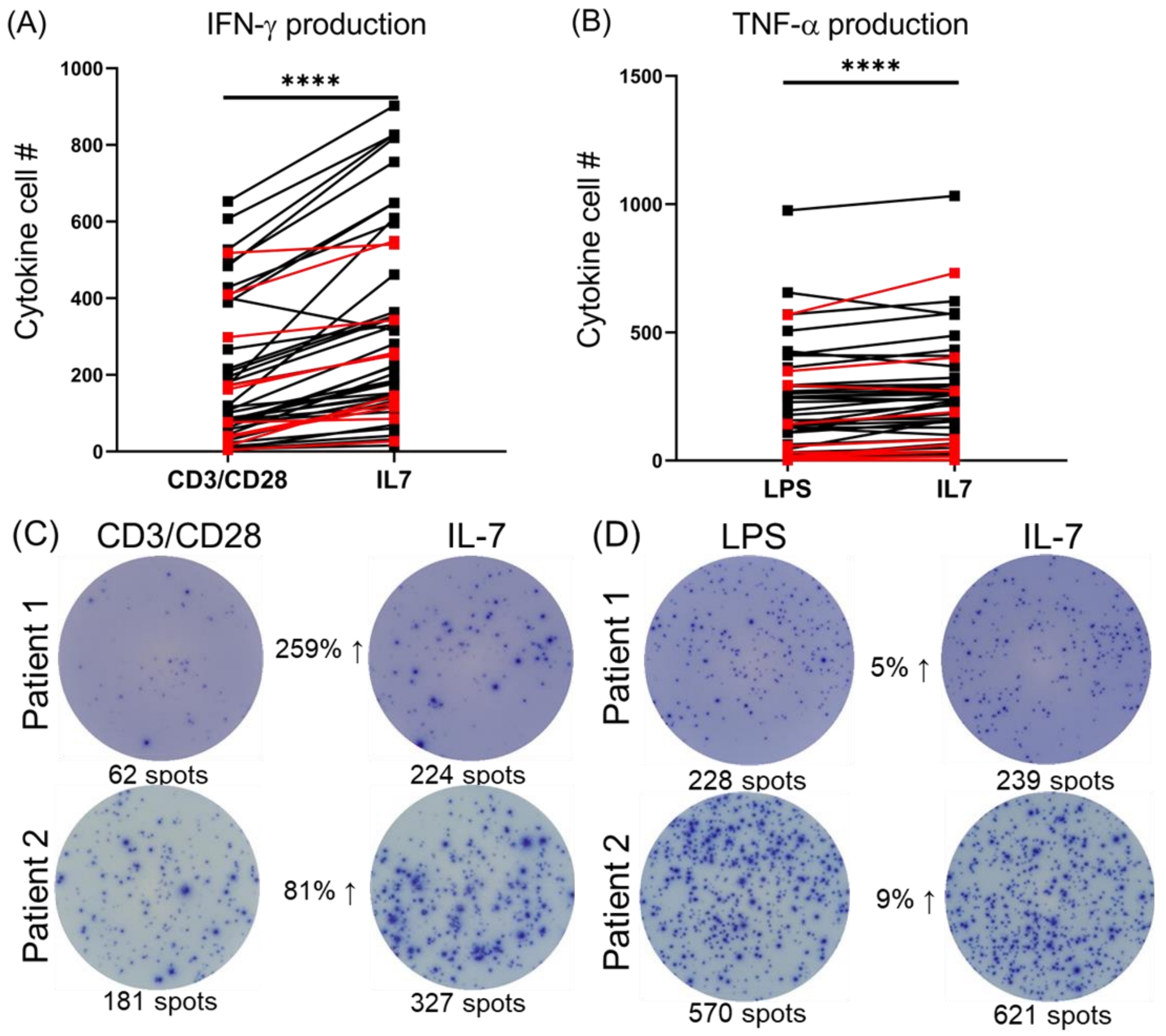

Supplemental Figure 4. IL-7 Effect on the innate and adaptive immune function in septic patients.

Line plot demonstrating change in number of cytokine producing cells using ELISpot between control (anti-CD3/anti-CD28 antibody or LPS) samples and stimulation with IL-7 for IFN-ɣ (A) and TNF-α (B). Panels (C), (D) are representative photomicrographs demonstrating ELISpot change from control sample to IL-7 stimulated for IFN-ɣ and TNF-α. Each dot represents an individual patient. Red lines represent patients who died. Paired samples were analyzed using a paired rank sum Wilcoxon test. IFN-ɣ n= 25, TNF-α n=25.

Supplemental Figure 5:

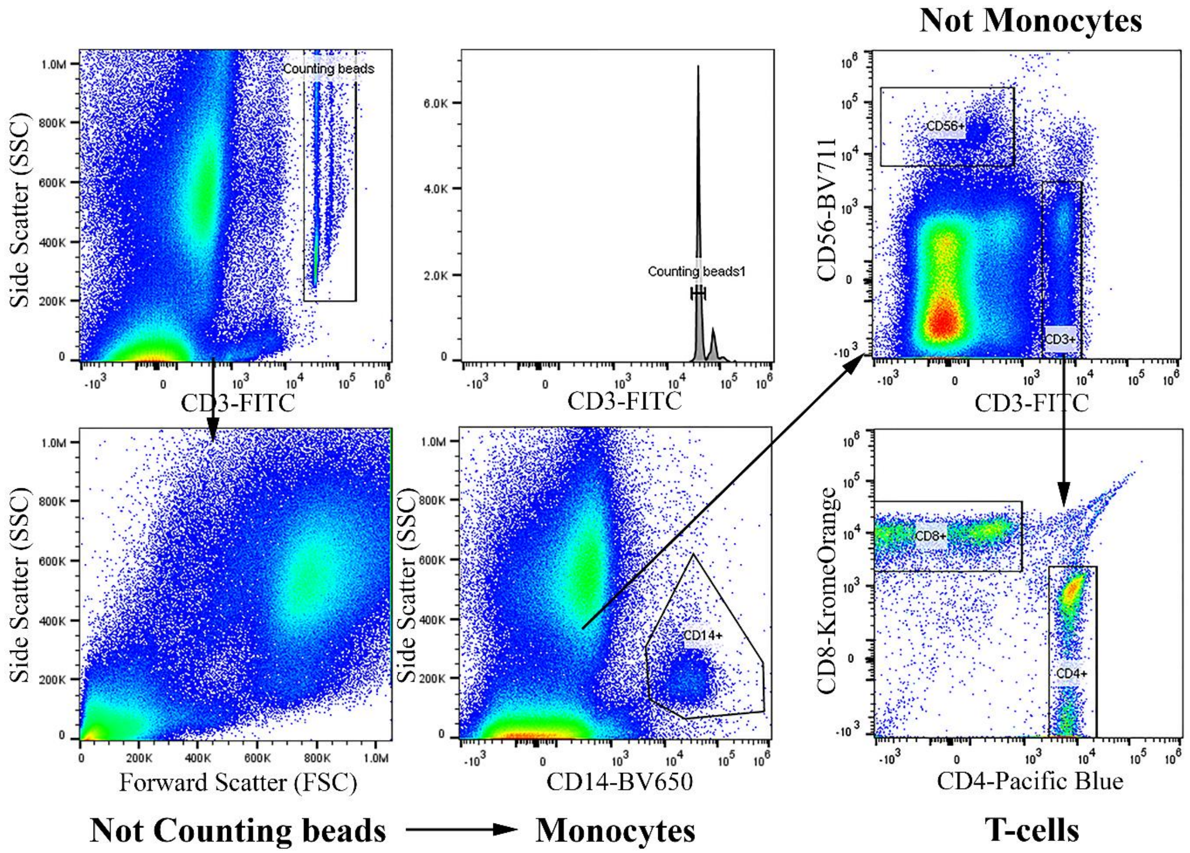

Supplemental Figure 5. LUCID DURA flow tube gating strategy.

Scatter plot for gating with FITC x SSC and histogram for singlets (counting beads1). All events that did not fall into the initial counting bead gate went through doublet discrimination (FSC-A x FSC-W, and then SSC-A x SSC-W) and were then examined for CD14 expression; these $CD14^+$ cells were used to calculate number of monocytes. All events that were not $CD14^+$ were then examined for $CD56^+$ positivity (Used to calculate number of NK cells) and CD3 positivity (Used to calculate number of $CD3^+$ T cells, $CD56^+CD3^+$ events were excluded). $CD3^+CD56^-$ events were then further interrogated for CD4 or CD8 single positivity. Absolute cell counts were determined using the counting beads according to manufacturer instructions.

Supplemental Figure 6:

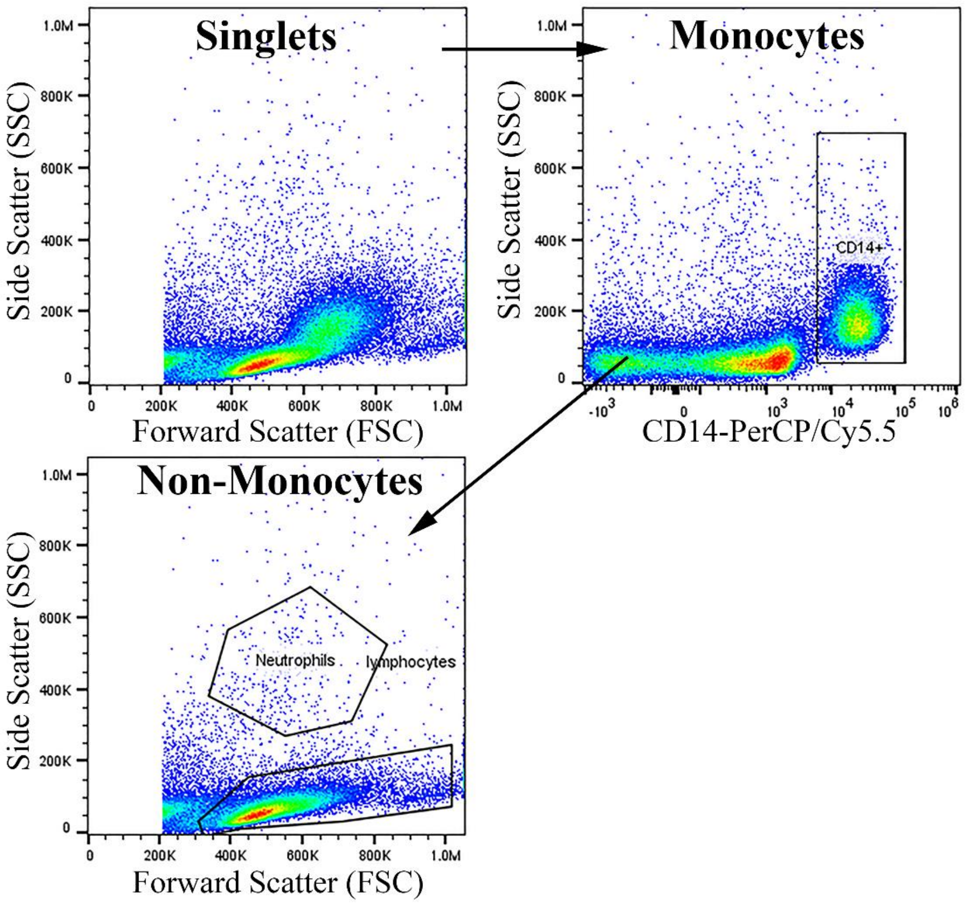

Supplemental Figure 6. PBMC gating strategy

Following doublet discrimination, PBMCs were gated for CD14 positivity. $CD14^{+}$ cells were considered monocytes. All events not falling into the $CD14^{+}$ gate were then examined on a FSC x SSC plot. Neutrophils and Lymphocytes were gated. Percentage of the whole singlet gate was then taken for use in determining cell number input in ELISpot assays.

Abbreviation	Explanation
PNA	Pneumonia
UTI	Urinary Tract Infection
MRSA	Methicillin-Resistant Staphylococcus Aureus
MSSA	Methicillin-Sensitive Staphylococcus Aureus
VAP	Ventilator-Associated Pneumonia
COVID-19	2019 Novel Coronavirus
CAP	Community-Acquired Pneumonia
ARDS	Acute Respiratory Distress Syndrome
SIRS	Systemic Inflammatory Response Syndrome
ARF	Acute Respiratory Failure
AHRF	Acute Hypoxemic Respiratory Failure
MVC	Motor Vehicle Crash

Supplemental Table 1

COVID-19 ICU Patients (n=27)			
**COVID-19 Patients**	**Age**	**Sex**	**Primary Diagnosis**
Patient 1	57	M	Acute hypoxemic respiratory failure secondary to adult respiratory distress syndrome due to COVID-19 pneumonia
Patient 2	57	F	CAP, viral pneumonia
Patient 3	40	F	Acute respiratory distress syndrome (ARDS) due to COVID-19 virus
Patient 4	67	M	Acute hypoxemic respiratory failure secondary to ARDS/COVID-19 pneumonia
Patient 5	47	M	acute hypoxemic respiratory failure/ARDS secondary to COVID-19
Patient 6	52	F	Pneumonia due to COVID-19 virus
Patient 7	29	F	ARDS, COVID19+, transaminitis, metabolic acidosis, lactic acidosis + starvation ketosis, hyponatremia
Patient 8	49	M	SIRS, Viral pneumonia vs bacterial, ARF
Patient 9	84	M	AHRF, COVID-19, ARDS,
Patient 10	69	F	AHRF, COVID19, transaminitis, acute kidney injury
Patient 11	25	M	Acute hypoxemic respiratory failure secondary to COVID-19 pneumonia
Patient 12	33	F	COVID-19
Patient 13	57	M	COVID-19 virus infection
Patient 14	61	M	Pneumonia due to COVID-19 virus
Patient 15	70	M	covid-19 viral pneumonia, acute hypoxemic resp failure
Patient 16	34	M	Acute hypoxemic respiratory failure due to ARDS, COVID-19 pneumonia
Patient 17	64	M	Pneumonia due to COVID-19 virus
Patient 18	79	M	Acute hypoxemic respiratory failure: ARDS due to COVID-19 pneumonia
Patient 19	68	F	respiratory failure secondary to COVID-19
Patient 20	62	F	Acute hypoxemic respiratory failure, COVID-19, sepsis, acute renal failure
Patient 21	67	M	acute hypoxemic respiratory failure/ARDS secondary to COVID-19
Patient 22	71	M	acute hypoxemic respiratory failure/ARDS secondary to COVID-19
Patient 23	51	F	acute hypoxemic respiratory failure/ARDS secondary to COVID-19
Patient 24	59	F	acute hypoxemic respiratory failure/ARDS secondary to COVID-19
Patient 25	64	F	acute hypoxemic respiratory failure/ARDS secondary to COVID-19
Patient 26	53	F	acute hypoxemic respiratory failure/ARDS secondary to COVID-19
Patient 27	86	M	acute hypoxemic respiratory failure/ARDS secondary to COVID-19

Supplemental Table 2

Septic Patients (n=51)					
Septic Subjects	Age	Sex	Primary Diagnosis	Source of Infection	Organism
Patient 1	36	F	Jaundice	PNA	Unknown
Patient 2	40	F	Necrotizing Soft Tissue Infection	Necrotizing Fasciitis	Streptococcus agalactiae
Patient 3	39	M	Small Bowel Obstruction	Peritonitis	Unknown
Patient 4	49	M	Motor Vehicle Collison	Wound Infection	Enterococcus faecium, Fusarium, Mycobacterium smegmatis, Exophiala
Patient 5	89	F	Mesenteric Ischemia	Peritonitis	Yeast, Klebsiella pneumoniae, Bacteroides fragilis group
Patient 6	54	M	Acute Respiratory Failure with Hypoxia	PNA	Influenza
Patient 7	73	F	Mesenteric Ischemia	Peritonitis	Unknown
Patient 8	69	F	Necrotizing Fasciitis	Necrotizing Fasciitis	Escherichia coli, Streptococcus anginosus, Bacteroides fragilis group
Patient 9	79	F	Motor Vehicle Collison	PNA	MRSA
Patient 10	31	F	Acute Encephalopathy	PNA	MSSA
Patient 11	58	F	Small Bowel Obstruction	Peritonitis	Unknown
Patient 12	22	M	Sacral Fracture Closed	UTI	Escherichia coli
Patient 13	54	F	Septic Shock	PNA	MRSA
Patient 14	26	M	Traumatic Brain Injury	PNA	MSSA
Patient 15	44	M	Bowel Perforation	Peritonitis	Escherichia coli, Streptococcus constellatus, Bacteroides fragilis group
Patient 16	33	F	Esophageal Perforation	Mediastinitis	Coagulase negative staphylococcus species, Candida albicans
Patient 17	75	M	Septic Shock - Urinary Tract Infection	UTI	Proteus mirabilis
Patient 18	54	M	Spinal Cord Compression	PNA	Yeast
Patient 19	39	M	Intraabdominal Fluid Collection	Peritonitis	Coagulase negative staphylococcus
Patient 20	62	F	Acute Hypoxemic Respiratory Failure	PNA	MSSA
Patient 21	40	M	Septic Shock	VAP	MSSA
Patient 22	33	M	Pneumoperitoneum	Peritonitis	Coagulase negative staphylococcus
Patient 23	57	M	Stroke	UTI	Enterobacter cloacae complex
Patient 24	79	M	Septic Shock	PNA	Streptococcus pneumoniae
Patient 25	18	F	Diffuse Axonal Brain Injury	PNA	MSSA
Patient 26	82	F	Acute Hypoxemic Respiratory Failure due to Multifocal Pneumonia	PNA	Unknown
Patient 27	54	F	Septic Shock	UTI	Pseudomonas Aeruginosa, Enterococcus Faecalis
Patient 28	83	F	Mixed Shock of Unknown Etiology	Unknown	Unknown
Patient 29	54	M	Intraparenchymal Hemorrhage	Peritonitis	Unknown
Patient 30	63	M	Weakness	Necrotizing Fasciitis	Enterobacter species
Patient 31	60	F	Decompensated Hepatic Cirrhosis	Unknown	Unknown
Patient 32	59	M	Acute Respiratory Failure with Hypoxia	PNA	Parainfluenza 3
Patient 33	72	M	Spontaneous Bacterial Peritonitis	Peritonitis	Other Staphylococcus species
Patient 34	59	M	Hepatic Cirrhosis	PNA	Haemophilus influenza
Patient 35	68	F	Respiratory Failure	PNA	Enterobacter species
Patient 36	46	M	Acute Liver Failure	Peritonitis	Other Staphylococcus species
Patient 37	66	F	GI Bleed	Wound Infection	Acinetobacter, Pseudomonas, Enterococcus faecium, Bacteroides fragilis, Stenotrophomonas maltophilia
Patient 38	57	M	Respiratory Failure	Peritonitis	Mixed gram positive microorganisms
Patient 39	78	F	Septic Shock	PNA	Escherichia coli, MRSA
Patient 40	62	F	Pneumatosis Coli	Peritonitis	Staphylococcus species
Patient 41	64	F	MSSA Bacteremia	Wound Infection	Staphylococcus aureus
Patient 42	72	M	Arterial Thromboembolism	Peritonitis	Unknown
Patient 43	62	F	Toxic Metabolic Encephalopathy	Peritonitis	Unknown
Patient 44	59	F	Acute on Chronic Respiratory Failure	Wound Infection	Staphylococcus epidermidis
Patient 45	28	F	Diabetic Ketoacidosis	PNA	Unknown
Patient 46	75	M	Septic Shock	PNA	Coronavirus HKU1 RNA
Patient 47	87	F	Sepsis due to Urinary Tract Infection	UTI	Enterobacter species
Patient 48	66	F	Perforated Diverticulum	Peritonitis	Unknown
Patient 49	59	M	Septic Shock	UTI	Escherichia coli
Patient 50	59	F	Septic Shock	PNA	Streptococcus species
Patient 51	37	M	Necrotizing Pancreatitis	UTI	Escherichia coli, Enterococcus faecalis

Supplemental Table 3

Critically Ill Non-Septic Patients (n=18)			
**Critically Ill Patients**	**Age**	**Sex**	**Primary Diagnosis**
Patient 1	57	M	Polytrauma, Skull Fracture and Subarachnoid Hemorrhage
Patient 2	36	M	Surgical Repair for Closed Fracture of Thyroid Cartilage
Patient 3	56	M	Pelvic Ring Fracture
Patient 4	64	F	Popliteal Arterial Occlusion, left
Patient 5	56	M	Motor Vehicle Collison
Patient 6	23	F	Desmoid Tumor Resection, paraspinal
Patient 7	40	M	Closed Non-displaced Fracture C6
Patient 8	61	M	Retroperitoneal Hematoma, distal aorta
Patient 9	80	M	Ruptured Abdominal Aortic Aneurysm
Patient 10	61	M	Motor Vehicle Collison
Patient 11	54	F	Lumbar Spinal Stenosis with Neurogenic Claudication
Patient 12	62	F	Arterial Occlusion
Patient 13	61	M	Ruptured Abdominal Aortic Aneurysm
Patient 14	70	F	Arterial Occlusion
Patient 15	62	M	Popliteal Arterial Occlusion, left
Patient 16	63	M	Arterial Occlusion, lower extremity
Patient 17	75	M	Central Cord Syndrome
Patient 18	77	F	Pulmonary Artery Saddle Embolus

Supplemental Table 4

Septic Total	51	
**Primary Diagnosis**		
Infectious disease	19	37%
Gastrointestinal disease	7	14%
Respiratory disease	7	14%
Trauma (MVC, Fractures)	5	10%
Liver Disease	4	8%
Neurologic disease	4	8%
Clotting/Bleeding disorder	2	4%
Metabolic disorder	1	2%
Spinal cord injury/disease	1	2%
Shock	1	2%
**Source of Infection**		
PNA/VAP	19	37%
Peritonitis	15	29%
UTI	7	14%
Wound Infection	4	8%
Necrotizing Fasciitis	3	6%
Unknown	2	4%
Mediastinitis	1	2%

Critically Ill Non-Septic Total	18	
**Primary Diagnosis**		
Trauma (MVC, Fractures)	6	33%
Peripheral vascular disease	5	28%
Spinal cord injury/disease	2	11%
Cardiovascular disease	2	11%
Clotting/Bleeding disorder	2	11%
Neoplasm (nonmalignant)	1	6%
